# Characterization of the Deep-Sea *Streptomyces* sp. SCSIO 02999 Derived VapC/VapB Toxin-Antitoxin System in *Escherichia coli*

**DOI:** 10.3390/toxins8070195

**Published:** 2016-07-01

**Authors:** Yunxue Guo, Jianyun Yao, Chenglong Sun, Zhongling Wen, Xiaoxue Wang

**Affiliations:** 1 Key Laboratory of Tropical Marine Bio-resources and Ecology, Guangdong Key Laboratory of Marine Materia Medica, Research Network for Applied Microbiology (RNAM) Center for Marine Microbiology, South China Sea Institute of Oceanology, Chinese Academy of Sciences, Guangzhou 510301, China; yaojianyun.yun@163.com (J.Y.); clsun@scsio.ac.cn (C.S.); zlwen@scsio.ac.cn (Z.W.); 2 University of Chinese Academy of Sciences, Beijing 100049, China

**Keywords:** toxin-antitoxin, VapC/VapB, deep sea, *Streptomyces*

## Abstract

Toxin-antitoxin (TA) systems are small genetic elements that are ubiquitous in prokaryotes. Most studies on TA systems have focused on commensal and pathogenic bacteria; yet very few studies have focused on TAs in marine bacteria, especially those isolated from a deep sea environment. Here, we characterized a type II VapC/VapB TA system from the deep-sea derived *Streptomyces* sp. SCSIO 02999. The VapC (virulence-associated protein) protein belongs to the PIN (PilT *N*-terminal) superfamily. Overproduction of VapC strongly inhibited cell growth and resulted in a bleb-containing morphology in *E. coli*. The toxicity of VapC was neutralized through direct protein–protein interaction by a small protein antitoxin VapB encoded by a neighboring gene*.* Antitoxin VapB alone or the VapB/VapC complex negatively regulated the *vapBC* promoter activity. We further revealed that three conserved Asp residues in the PIN domain were essential for the toxic effect of VapC. Additionally, the VapC/VapB TA system stabilized plasmid in *E. coli*. Furthermore, VapC cross-activated transcription of several TA operons via a partially Lon-dependent mechanism in *E. coli*, and the activated toxins accumulated more preferentially than their antitoxin partners. Collectively, we identified and characterized a new deep sea TA system in the deep sea *Streptomyces* sp. and demonstrated that the VapC toxin in this system can cross-activate TA operons in *E. coli*.

## 1. Introduction

Toxin-antitoxin (TA) systems are widely distributed in archaea and bacteria. Recent studies have proven that activated toxins inhibit essential cellular processes, including DNA replication [1], mRNA stability [2], translation [3], cytoskeleton formation [4], membrane integrity [5,6] and cell wall synthesis [7]. TA-elicited alteration of cellular processes results in important physiological changes, such as the formation of metabolically dormant cells [8,9,10], higher tolerance to antibiotics [11], and increased phage inhibition [12]. Based on the nature and mode of action of antitoxin, TA systems have been divided into five different types [4,5,13,14]. The toxin components in TAs are all small proteins, while the antitoxins are either labile proteins or untranslated antisense RNAs. In type I TA systems, antitoxins bind to 5′ untranslated regions (UTR) or coding regions of toxin mRNAs through a complementary mechanism, and resulting in translation inhibition or degradation of toxin transcripts. In type II systems, antitoxin proteins bind to toxin proteins directly and inhibit the bioactivity of the toxins. In type III TA systems, an RNA antitoxin directly interacts with the toxin protein. Different from type I to type III TA systems, the components of type IV TA systems do not interact with each other, but instead, they have the same cellular target and the antitoxin suppresses the toxicity of toxin by stabilizing its target [4]. In the type V system, the antitoxin GhoS protein specifically cleaves the mRNA of the toxin *ghoT*, and thus prevents the translation of the toxin [5].

Type II TA systems have been the most extensively studied in the past, in part due to their abundance in bacterial genomes [15] and the development of bioinformatics tools to locate these loci in the sequenced genome based on their genetic features [16,17]. Currently, about 39 TA systems have been identified in *E. coli* K-12, including 18 type I, 19 type II, one type IV and one type V [4,5,18,19]. The type II toxins are RNases or DNA gyrase inhibitors [2,20,21,22,23,24,25,26]. Type II TA loci have been further classified into evolutionary independent gene families according to similarities of the toxins at the amino acid sequence level [27,28]. Among these families, VapC/VapB is the most common and represents more than 30% of all TA systems [16,27,29,30]. In *Mycobacterium tuberculosis*, 45 out of 88 type II TA loci are *vapBC* homologs [31]. However, in *E. coli* K-12, VapC/VapB homologs have not been identified.

Previous studies in TA systems have mostly focused on commensal and pathogenic bacteria. The marine ecosystem represents the largest ecosystem on earth and harbors the highest abundance and diversity of microorganisms [32]. Recently, the similarities and differences of core genes of the microbiomes between the marine ecosystems and the human gut have been compared using 243 ocean microbiome samples of the *Tara* Oceans Project [33,34] and microbiome samples from the human gut [35]. Despite large physicochemical differences between the two ecosystems, most of the prokaryotic gene abundance (73% in the ocean; 63% in the gut) can be attributed to a shared functional core [34]. Differences in the core gene abundance between the two ecosystems have also been revealed, including those involved with defense mechanisms, signal transduction, and energy production [34]. However, functional studies of TA systems in marine bacteria have been rarely explored. *Streptomyces* sp. SCSIO 02999 (SCSIO 02999) was isolated from South China Sea sediment at a depth of 880 m [36], and has been found to produce a variety of biologically active compounds with antivirus, antitumor or antibacterial activities [36,37]. The taxonomy of the strain was analyzed based on sequence of a 16S rRNA gene (GenBank accession No. JQ815089), and it is close to *Streptomyces* sp. VTT E-062988, ACT-40 and 1A01691. Here, we searched the genome of SCSIO 02999 and demonstrated that two neighboring genes (*00087* and *00088*) encode a type II VapC/VapB TA system. We further reveal that the VapC/VapB TA system confers plasmid stability and that the toxin VapC cross-actives many TA operons via a partially Lon-dependent mechanism in *E. coli*. To the best of our knowledge, this is the first report on characterizing a TA system in deep sea microorganisms.

## 2. Results

### 2.1. Identification of VapC/VapB TA

**Figure 1:**
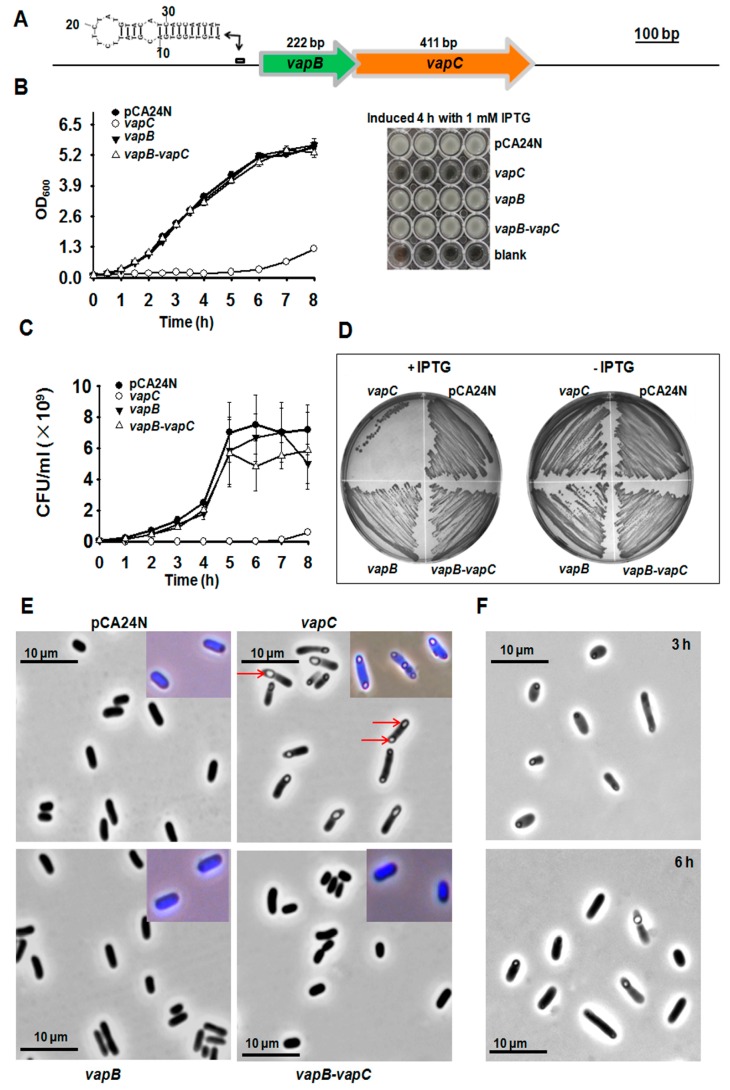
VapC (00087) is toxic and VapB (00088) neutralizes the toxicity of VapC. (**A**) Chromosomal loci of *vapBC* operon. The secondary structure of the palindrome near the Ribosomal Binding Site (RBS) is also shown. (**B**) Growth of *E. coli* K-12 BW25113 harboring pCA24N-based constructs that were induced with 0.5 mM IPTG at OD_600_ 0.1. Cell growth was tested at the time points indicated (left panel) and images were taken after 4 h induction (right panel). (**C**) Cell viability (CFU/mL) were determined at the time points indicated. (**D**) *E. coli* hosts harboring the above-mentioned plasmids were streaked onto LB plates supplemented with 30 μg/mL chloramphenicol with or without 0.5 mM IPTG, and were incubated for 16 h. (**E**) Morphology of BW25113 cells overproducing VapC, VapB and VapB-VapC TA. Red arrows point to the “blebs” of cells. Cells were grown in LB and induced with 0.5 mM IPTG at OD_600_ 1.0 for 3 h. Induced cells stained with 4’, 6-Diamidino-2-Phenylindole (DAPI) were shown in the right corners. (**F**) BW25113 cells in (**E**) were washed with PBS to remove isopropyl beta-d-thiogalactopyranoside (IPTG) and re-cultured for another 3 h and 6 h, respectively. Data are from three independent cultures and standard deviations are shown in (**B**) and (**C**). At least two independent cultures were used and representative images were shown in (**D**–**F)**.

The putative TA loci in the deep sea *Streptomyces* sp. SCSIO 02999 genome were predicted with a web based tool RASTA-Bacteria [17], and several potential TA pairs were identified. Two neighboring genes, *orf00087* and *orf00088*, encode two small proteins of 136 aa and 73 aa, respectively (Figure 1A and Figure S1); Orf00087 was predicted to be homologous to the toxin VapC of the VapC/VapB type II TA pair (Figure S2). To test the toxicity of the two-gene cassette, we cloned the coding regions into the pCA24N plasmid with a *lac* promoter to construct pCA24N-*orf00087* (pCA24N-*vapC*) and pCA24N-*orf00088* (pCA24N-*vapB*) using genomic DNA of *Streptomyces* sp. SCSIO 02999 as the template. After transformation into *E. coli* K-12 BW25113 and induction with isopropyl beta-d-thiogalactopyranoside (IPTG), cells expressing VapC using pCA24N-*vapC* exhibited a notable decrease in cell growth as shown by the reduction in turbidity at 600 nm (OD_600_) and colony forming units (CFUs) (Figure 1B–D). In contrast, overexpression of VapB using pCA24N-*vapB* did not affect cell growth (Figure 1B–D). Phase-contrast microscopic examination revealed that expression of the toxin led to formation of morphologically altered, non-replicating “bleb-containing” cells in *E. coli*. Almost all of the cells contained one to three “blebs” in the middle or near the poles (Figure 1E). The 4’, 6-Diamidino-2-Phenylindole (DAPI) staining showed that the “blebs” did not contain nucleoids (Figure 1E), and these membrane protrusions might be formed by the membrane damage generated in the cytoplasm when toxin VapC was overproduced. When IPTG was removed from cells overexpressing VapC, the size of the “blebs” was reduced in 3 h (Figure 1F, upper panel) and the number of cells having “blebs” greatly decreased in 6 h (Figure 1F, lower panel).

To determine whether the neighboring protein VapB can neutralize the toxicity of VapC, we constructed the pCA24N-*00088*-*00087* (pCA24N-*vapB*-*vapC*) plasmid to co-express VapB and VapC in *E. coli* cells. As expected, VapB completely neutralized the toxicity of VapC (Figure 1B–D). Moreover, co-expression of VapB effectively inhibited the formation of blebs caused by VapC overproduction (Figure 1E). Thus, expression of VapC from the deep sea *Streptomyces* sp. resulted in growth inhibition of *E. coli*, and VapB serves as the antitoxin partner for VapC.

### 2.2. VapB and VapC Form a Complex in Vivo

**Figure 2:**
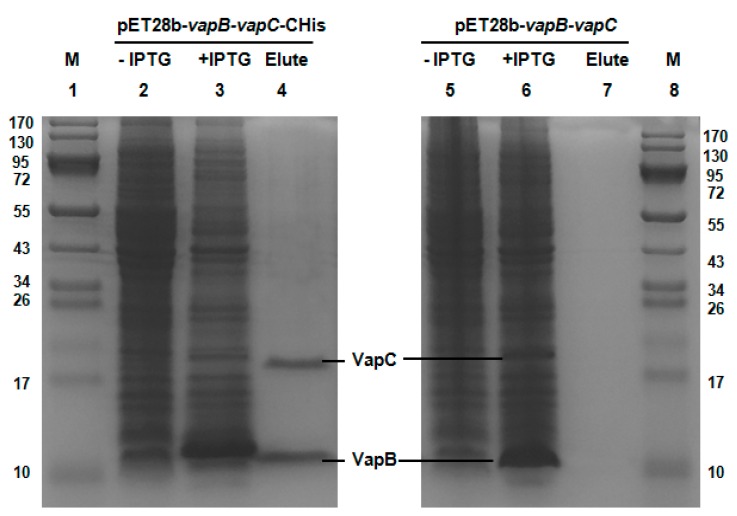
VapC and VapB form a complex in vivo. Plasmid pET28b-*vapB*-*vapC-*Chis was constructed to produce a His-tagged VapC and untagged VapB with IPTG induction, 15.74 kDa VapC-Chis and 8.59 kDa VapB were induced (lane 3). During purification, VapB was co-purified (lane 4). Cells that were not induced with IPTG were served as control (lane 2). Additionally, as a negative control (lane 5–7), pET28b-*vapB*-*vapC* was also constructed to produce untagged VapB and untagged VapC, and neither VapB nor VapC bound to the Ni-NTA agarose beads (lane 7). The protein marker (M) was loaded in lanes 1 and 8.

A typical feature of type II TA systems is that the toxin interacts with antitoxin directly and they form a protein complex in vivo [38]. Here, we performed a pull-down assay using pET28b-*vapB*-*vapC*-Chis to express a *C*-terminal hexahistidine tagged (His-tagged) VapC along with an untagged antitoxin VapB. Affinity purification using Ni-NTA agarose beads and subsequent Tricine-SDS-PAGE revealed that a small protein could be pulled down together with His-tagged VapC (Figure 2, Lane 2–4), and this small protein was found to be VapB by mass spectrometry. As a control, we constructed a pET28b-*vapB*-*vapC* to express untagged VapB and untagged VapC, and neither of them could bind to Ni-NTA beads (Figure 2, lane 5–7). Hence, we show that VapB interacts with VapC and they formed a complex in vivo.

### 2.3. VapB and VapC/VapB Negatively Autoregulate the VapBC Operon

**Figure 3:**
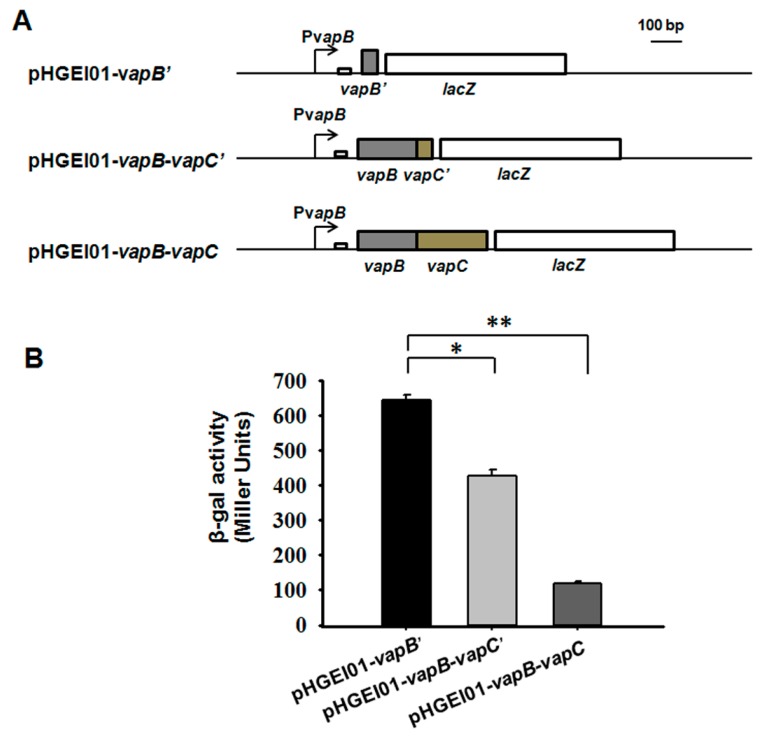
VapB and VapB-VapC complex both regulate *vapBC* operon. (**A**) Schematic diagram of the constructed reporter systems for the promoter activity assay. (**B**) Mid-log-phase *E. coli* WM3064 cells harboring the reporter systems in (**A**) were collected and tested for β-galactosidase activity. Three independent cultures for each strain were used and the data are shown as means ± standard deviations. Asterisks represent a statistically significant difference (*p* < 0.01 was shown in * and *p* < 0.001 was shown in **; *n* = 3).

An in vivo promoter activity assay was used to study the autoregulation of the *vapBC* operon. We amplified three different fragments containing a 260 bp upstream region of *vapBC* followed by the full coding region of *vapBC* (*vapB*-*vapC*), the full coding region of VapB followed by the first 45 bp of the coding region of VapC (*vapB*-*vapC’*), and the first 45 bp of the coding region of VapB (*vapB’*). These three fragments were fused to the *lacZ* gene and ligated into the pHGEI01 plasmid (Figure 3A). The promoter activity was decreased from 645.0 ± 13.6 miller units (MU) in *E. coli* WM3064 cells carrying the pHGEI01-v*apB’* plasmid to 426.3 ± 17.9 MU in cells harboring the pHGEI01-v*apB-vapC’*(Figure 3B), suggesting that the presence of VapB repressed the promoter activity. Moreover, the cells carrying the pHGEI01-v*apB-vapC* showed much lower promoter activity (117.8 ± 7.5 MU), suggesting that the VapC helped the VapB to repress the promoter activity. One palindrome of 14 bp near the ribosome binding site (RBS) was found (Figure 3A and Figure S1), probably serving as the binding site for VapB since most type II antitoxins bind to DNA at palindromic regions.

### 2.4. Key Residues for the Toxicity of VapC

**Figure 4:**
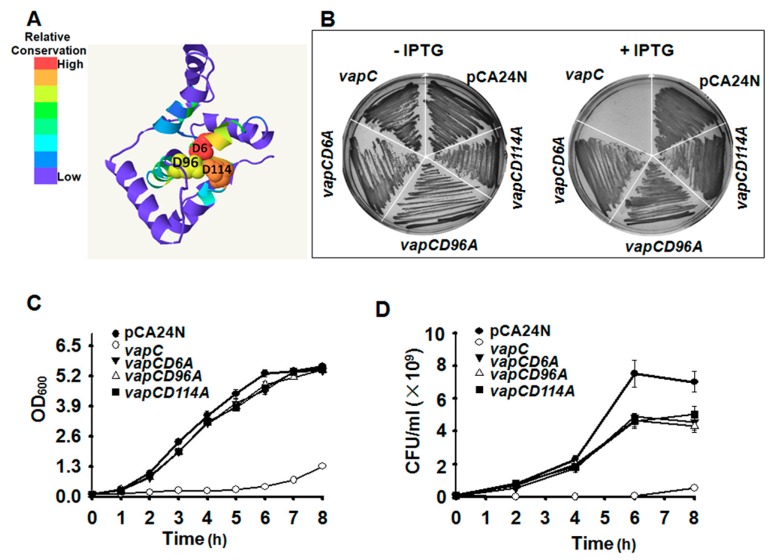
Key residues for determining VapC toxicity. (**A**) Predicted 3D structure of VapC. The three conserved D in PilT *N*-terminal (PIN) domain were indicated. The number in the mutated protein indicates the position of the amino acid in VapC. (**B**) Toxicity after single-site mutagenesis to convert the three D residues to A was determined in *E. coli* BW25113 wild type. Growth (**C**) and colony forming units (CFU) (**D**) tests of *E. coli* cells expressing the VapC and the three mutant VapC proteins, 0.5 mM IPTG was added at OD_600_ 0.1. Three independent cultures were evaluated for each analysis, and only one representative image is shown in (**B**).

Phyre2 [39] was applied to predict the 3D structure of VapC (Figure 4A), which belongs to PIN (PilT *N*-terminal) domain superfamily. Proteins of the PIN superfamily are small RNases. Three strictly conserved acidic residues (Asp, D) commonly found in the RNase active site of PIN domain were also identified in VapC (at position 6, 96 and 114) (Figure 4A). To investigate the importance of the conserved acidic residues in determining the toxicity of VapC, we performed site-directed mutagenesis on the D residues at positions 6, 96 and 114, separately. The results suggested that any mutation of the three D residues completely abolished the toxicity of VapC (Figure 4B–D). The 3D structure predicted here indicated that the conserved active site formed a negatively charged pocket near the center of the molecule, and they might be essential for single-stranded ribonuclease activity (Figure 4A). Furthermore, the three mutated VapCs no longer induced “bleb-containing” cells (Figure S3), further confirming the key role of the three residues in determining VapC toxicity.

### 2.5. VapC/VapB Stabilizes Plasmids in E. coli

**Figure 5:**
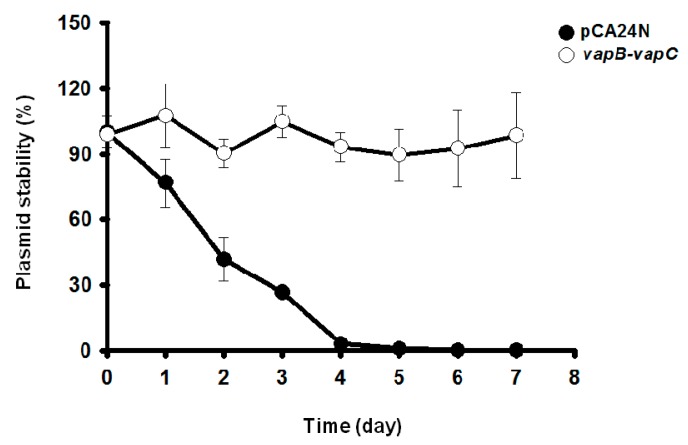
VapC/VapB toxin-antitoxin (TA) system confers plasmid stability in *E. coli*. *E. coli* K-12 BW25113 harboring plasmids pCA24N and pCA24N-*vapB-vapC* were used for the plasmid stability assay. Overnight cultures were diluted 100-fold in LB medium without any antibiotics, then incubated at 37 °C for 12 h. This process was repeated every 12 h for seven days. Three independent cultures were conducted, and the data are shown as means ± standard deviations.

Toxin-antitoxin systems are well known to stabilize plasmids in cells. The ability of VapC/VapB to stabilize plasmids was tested in *E. coli*. Empty plasmid pCA24N carrying a chloramphenicol resistance gene exhibited a loss of ~99% in *E. coli* in the absence of chloramphenicol after continuous culture for four days. In contrast, cells expressing TA pair VapB/VapC did not exhibit significant plasmid loss (Figure 5). The *E. coli* cells harboring pCA24N completely lost resistance to chloramphenicol after five days, whereas more than 90% of the cells harboring the pCA24N-*vapB*-*vapC* were still resistant to chloramphenicol after seven days (Figure 5). These results demonstrate that the VapC/VapB TA pair from the deep sea *Streptomyces* sp. SCSIO 02999 confers plasmid stabilization in *E. coli.*

### 2.6. VapC Cross-Activates E. coli TA Systems in a Partially Lon-Dependent Manner

**Figure 6:**
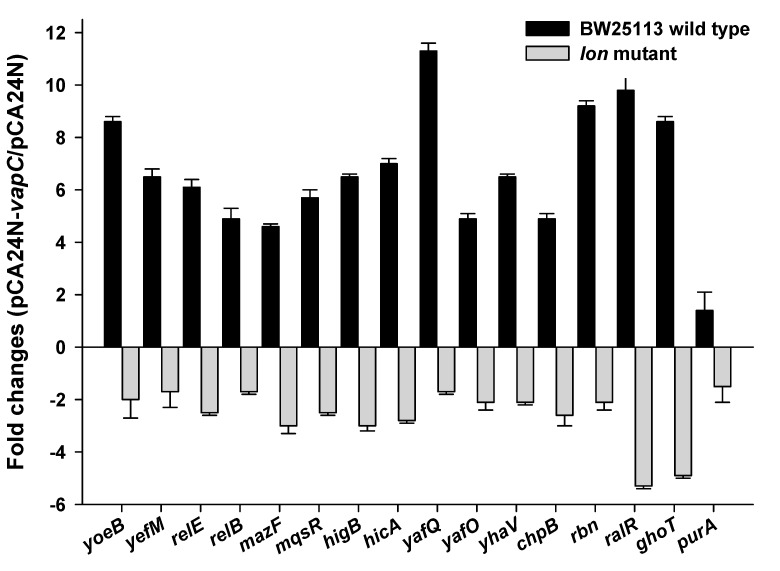
VapC cross-activates the toxins in *E. coli* in a Lon-dependent manner. Fold changes of 14 TA transcripts and 1 RNase gene (*rbn*) in cells overexpressing VapC via pCA24N-*vapC* as compared to empty vector pCA24N in *E. coli* were quantified by qRT-PCR. The *purA* was used as negative control. Two independent cultures were used for the assay, and standard errors are indicated.

Cross-talk among TAs has been reported previously [40,41]. VapC-VapB in the deep sea microbe could be transferred into a different host if it is present on a mobile genetic element such as conjugative plasmids. Here, we explored whether deep sea derived VapC also activates transcription of reported TA systems, especially type II endoribonucleases in *E. coli*. The VapC was overexpressed via pCA24N-*vapC* after IPTG induction for 1 h in *E. coli* K-12 host. Changes at the transcriptional levels of toxin components of ten different type II TAs (the same 10 TA deleted in the Δ10 strain [42]) and two other toxins (RalR of type I TA and GhoT of type V TA) [5,19] were determined via qRT-PCR. Unexpectedly, transcription of all twelve toxins was induced by VapC overproduction (Figure 6). To check whether the antitoxins that are co-transcribed with the toxin components were also induced, expression levels of *yefM* and *relB* were also determined. The expression levels of these two antitoxins were both induced, but with lower fold-changes compared to their toxin components (Figure 6). VapC also induced the expression of another RNase gene *rbn* (Figure 6). As a negative control, expression of *purA* (adenylosuccinate synthase gene) was not affected by VapC overproduction (Figure 6). Moreover, the upregulation of toxin transcriptions by VapC was partially abolished in a Δ*lon* mutant strain (Figure 6), suggesting that the cross-activation of TA systems by VapC functions is partially Lon-dependent.

## 3. Discussion

Our findings strongly support the hypothesis that VapC and VapB in deep sea *Streptomyces* sp. SCSIO 02999 form a type II TA pair. The evidences are as follows: (i) both proteins are small; (ii) VapC is a toxin that inhibits cell growth and induces a bleb-containing phenotype in *E. coli*; (iii) the cognate VapB counteracts the toxicity of VapC through direct protein–protein interaction; (iv) the antitoxin VapB and VapB-VapC complex negatively regulates promoter activity of the *vapBC* operon; (v) the VapC/VapB TA stabilizes plasmid in *E. coli*; and (vi) ectopic production of VapC cross-activates many TA operons via a partially Lon-dependent mechanism in *E. coli*. These observations fit the features of type II TA system.

The VapC toxin identified in SCSIO 02999 belongs to the PIN domain (PF01850) superfamily, which contains 3673 proteins from 721 different species across the three domains of life [43]. Based on the secondary structure, 130 residues of the deep sea VapC (96% coverage) shared the highest similarity with VapC from *Mycobacterium tuberculosis* [44] (Figure S2). In eukaryotes, PIN domains are found in proteins involved in nonsense mediated mRNA decay [45], and in processing of 18S ribosomal RNA [46]. The majority of PIN-domain containing proteins identified in prokaryotes encodes the VapC toxins of VapC/VapB TA systems [47,48]. Recently, the PIN domain was identified in the ocean microbes by hologenome analysis of marine sponges microbiomes [49]. The VapC proteins are RNases targeting cellular mRNAs or tRNA^fMet^, showing substrates sequence specific or non-specific activities [50,51,52,53,54,55]. The cell morphology induced by VapC overproduction is different from that induced by other known toxins in *E. coli*. For example, the “ghost” cells were caused by overproduction of the lytic membrane toxin GhoT of type V GhoT/GhoS TA pair [5], the “filamentous growing” cells were induced by the toxin ParE of ParE/ParD TA pair [56,57], and the “swollen” cells were induced by higher eukaryotes and prokaryotes nucleotide-binding (HEPN) family toxin [58]. These results suggest that VapC in SCSIO 02999 should have different cellular targets from other known toxins in *E. coli*. Additionally, the “ovoid cells” morphology was reported for VapC overproduction in *Mycobacterium smegmatis* [59]. These previous results indicate that VapC from different species may have different targets and may be involved in distinct biological processes. Thus, the marine derived TAs may have potential application for treating pathogenic bacteria infection due to their similarity and differences to those in commensal and pathogenic bacteria.

Structural analysis has revealed that the antitoxin VapB tightly wrapped around toxin VapC to neutralize its toxicity [50,60,61]. Four DNA binding domains in VapB have also been characterized previously, including helix–turn–helix (HTH), ribbon–helix–helix (RHH), AbrB, and Phd/YefM domains [28,62]. VapB of SCSIO 02999 shares a similar RHH domain with VapB3 in *M. Tuberculosis,* and we demonstrated that both VapB and VapB-VapC are capable of auto-regulating the transcription of the TA operon. In this study, the attempt to purify the VapC was unsuccessful due to its high toxicity, which is consistent with previous studies [63,64]. In addition, co-purification of VapB and VapC resulted in a strong interaction between VapB and VapC, which made it difficult to separate them without affecting the activity of the toxin. New approaches are in needed to purify the toxin for in vitro characterization of the cellular targets of the toxin.

Studies on cross-activation among TA systems or other toxins mainly focus on type II TA system in which the toxin components are endoribonucleases. The well-studied type II TA MqsR/MqsA induces or represses various toxin genes in *E. coli*. For example, titrating antitoxin MqsA with MqsR or degrading MqsA through proteases Lon and ClpXP represses the expression of small toxic gene *cspD* [65]. In contrast, the deletion of toxin gene *mqsR* represses small toxic polypeptides encoding genes *hokA* and *hokE* [65]. In addition, MqsR overproduction induced expression of the *relBEF* operon and *relF* encodes a *hok*-like toxin targeting the inner membrane [65,66]. Furthermore, MqsR degrades *ghoS* mRNA and enriches toxin *ghoT* mRNA of the GhoT/GhoS type V TA pair during stress conditions [40]. In turn, HipA toxin activates the *mqsR/mqsA* operon [22], and RelE toxin induces the transcription of several TA operons including *mqsR/mqsA* [66,67]. Expression of VapC from *Salmonella* and *Shigella* activates toxin gene *yoeB* expression in *E. coli*, and the activation depends on Lon protease [41]. However, cross-activation among TAs in *E. coli* can also occur in protease deficient strains such as in *lon*, *ppk*, *clpP*, and *hslV* deficient strains [66]. Collectively, these results suggest that cross-talk among TAs is rather complex, and the exact mechanism remains to be elucidated.

## 4. Experimental Procedures

### 4.1. Bacterial Strains, Plasmids and Growth Conditions

The *Streptomyces* sp. SCSIO 02999 and *E. coli* strains and plasmids used in this study are listed in Table 1, and the sequences of all primers used in this study are listed in Table S1. The *E. coli* strains were grown in luria-bertani (LB) medium at 37 °C. The marine-derived *Streptomyces* sp. SCSIO 02999 was isolated from a South China Sea sediment (E 109° 153.171′, N 116° 103.576′) at a depth of 880 m [36], the strain was grown in AM6 culture [37] at 28 °C, and the strain was deposited in the type culture collection of the Center for Marine Microbiology, Research Network of Applied Microbiology, South China Sea Institute of Oceanology, Chinese Academy of Sciences, Guangzhou, China. The taxonomy of the strain was analyzed based on a 16S rRNA gene and was deposited in GenBank (accession No. JQ815089) Chloramphenicol (30 µg/mL) was used for maintaining the pCA24N based plasmids, and kanamycin (50 µg/mL) was used for maintaining pET28b and pHGEI01 based plasmids.

### 4.2. Cloning of Genes

The pCA24N-based plasmids were constructed according to a previous procedure [69] using primer pairs pCA24N-*vapB*-f/r, pCA24N-*vapC*-f/r and pCA24N-*vapB*-f/pCA24N-*vapC*-r. For pET28b-based constructs, different fragments were amplified with primer pairs pET28b-*vapB-*f/pET28b-*vapC-*Chis-r and pET28b-*vapB*-f/pET28b-*vapC*-r. After digestion with *Nco*I and *Hind*III, they were ligated into the pET28b empty vectors digested with the same two enzymes digested. The pHGEI01-based plasmids were constructed according to previous procedure [70] using primer pairs pHGEI01-P*vapB-vapC*-f as the forward primer, pHGEI01-*vapB*’-r1, pHGEI01-*vapC*’-r1 and pHGEI01-*vapB-vapC*-r1 as the trans-primer, respectively, for the first-step PCR; and then add the RBS (AGATCTCACACAGGAAACAGCT) sequence between the *vap* genes and the *lacZ* gene using primer pairs pHGEI01-P*vapB-vapC*-f and the primer pHGEI01-*vapB-vapC*-r2. The PCR products of the second-step PCR was digested with *EcoR*I and *BamH*I, the purified fragments were ligated into the plasmid digested with the same enzymes. All the three pHGEI01 based constructs are transcriptional fusion other than translational fusion. Genomic DNAs isolated from *Streptomyces* sp. SCSIO 02999 was used as DNA templates.

### 4.3. Protein Expression and Purification

The VapB-VapC complex containing a six histidine tag at the C-terminus of VapC and the VapB-VapC complex without any tag were purified via BL21 (DE3) with pET28b-*vapB-vapC*-CHis and pET28b-*vapB-vapC*, respectively. The strains were induced with 0.5 mM IPTG at OD_600_ 0.5 for 6 h. Then, the cells were collected and resuspended in 10 mL of lysis buffer (50 mM monosodium phosphate buffer (pH 8.0), 300 mM NaCl, 5 mM imidazole and protease inhibitor cocktail (Sigma-Aldrich, Shanghai, China)). The samples were lysed with the constant systems cell disruptor (Constant Systems Limited, Northants, UK) twice with 30 MPa. Nickel-nitrilotriacetic acid (Ni-NTA) agarose beads (Qiagen, Valencia, CA, USA) were used according to the manufacturer’s protocol. Purified proteins were desalted using a desalination column with 20 mM Tris-HCl buffer containing 300 mM NaCl (pH 8.0), and the protein concentration was measured using a Bi Yuntian bicinchoninic acid (BCA) assay kit (Bioteke corporation, Haimen, Jiangsu, China). Tricine-SDS-PAGE was performed as previously described [71]. A total of 20 µg of protein from each sample was loaded for SDS-PAGE.

### 4.4. DAPI (4′, 6-Diamidino-2-Phenylindole) Staining

Overnight cultures of *E. coli* K-12 BW25113 harboring pCA24N, pCA24N-*vapB*, pCA24N-*vapC* and pCA24N-*vapB-vapC* were diluted to OD_600_ 0.1, and cultured at 37 °C till the turbidity reached around 1.0. Then, 0.5 mM IPTG was added to induce protein expression for 5 h. Cells (2 × 10^5^ to 1 × 10^6^) were collected by centrifugation at 3000 g for 5 min, and the cell pellets were resuspended in 1 mL of phosphate buffer saline (PBS, pH 7.4). DAPI staining solution (300 µL of 300 nM) was added to the cell suspensions, then the cells were incubated for 5 min and rinsed several times in PBS before re-suspending in 100 µL PBS.

### 4.5. Promoter Activity Assay

Three plasmids were constructed to study the auto-regulation of the antitoxin VapB and VapB-VapC complex in vivo. A DNA fragment containing 260 bp upstream of the translational start site of *vapB* was selected as the promoter region, and pHGEI01-*vapB*’ contains the promoter region and the first 45 bp coding region of VapB, pHGEI01-*vapB*-*vapC’* contains the promoter region, the VapB coding region and the first 45 bp coding region of VapC, and pHGEI01-*vapB-vapC* contains the promoter region and full length *vapBC* operon. All three fragments were digested with *EcoR*I and *BamH*I and cloned into the promoter-less *lacZ*-fusion vector pHGEI01diegested with the two enzymes [71] to create plasmid pHGEI01-*vapB*’, pHGEI01-*vapB-vapC’* and pHGEI01-*vapB-vapC*. The resulting plasmids were verified by sequencing in WM3064. Mid-log-phase (OD_600_ ~ 0.7) cells of the indicated strains carrying the reporter systems were collected by centrifugation and washed with PBS. The β-galactosidase activity was measured according to previous protocols [72].

### 4.6. Site-Directed Mutagenesis

Single site-directed mutagenesis [2] was used to mutate the three conserved putative active sites of VapC. Mutation of D (GAT) to A (GCT) used primer pair *vapC*D6A-f/-r, D (GAC) to A (GCC) used primer pair *vapC*D96A-f/-r and D (GAT) to A (GCT) used primer pair *vapC*D114A-f/-r (Table S1). The mutations were verified by DNA sequencing using primers pCA24N-f.

### 4.7. Plasmid Stabilization Test

Overnight cultures of *E. coli* BW25113 carrying the plasmids pCA24N and pCA24N-*vapB-vapC* were obtained with chloramphenicol selection in LB. The cultures were diluted 1% in LB medium without antibiotics and cultured for 12 h. The cells were re-inoculated into 3 mL of fresh LB without antibiotics for a further 12 h is process. The cultures were serially diluted 10^0^–10^7^ by 10-fold from days 1 to 7, and 10 µL was dropped onto LB plates with and without 30 µg/mL of chloramphenicol. The plates were incubated at 37 °C for 16 h, and viable colonies were counted and the CFU were analyzed. The ratio of the chloramphenicol resistance colonies versus the total viable colony counts was used to estimate the percentage of plasmid maintained in the population. The CFU assay was conducted every day up to 7 days.

### 4.8. RNA Isolation and qRT-PCR

Total RNAs was isolated as indicated previously [73] and to avoid contamination of DNA to during the isolation process, DNase was added to treat the RNA samples for 30 min at room temperature. Then, the isolated RNAs were used as the templates for qRT-PCR reactions using the SuperScript^TM^ III Platinum SYBR^®^ Green One-Step qRT-PCR Kit (Invitrogen, Carlsbad, CA, USA). All of the primers for qRT-PCR are listed in Table S1. The level of the *rrsG* transcript was used as a reference to normalize the gene expression data. Exponentially growing cells (OD_600_ 0.8) were induced with 0.5 mM IPTG for 1 h. Lower Ct values indicate higher expression levels. Fold changes in the transcription of various targets with pCA24N or pCA24N-*vapC* were calculated as: 2^-((Ct *target*(pCA24N-*vapC*)-Ct*rrsG*(pCA24N-*vapC*)) -(Ct*target*(pCA24N)-Ct*rrsG*(pCA24N)))^.

## Figures and Tables

**Table 1:** Bacterial strains and plasmids used in this study. Cm^R^ and Km^R^ indicate chloramphenicol and kanamycin resistance, respectively. The aa indicates amino acids.

Bacterial Strains/Plasmids Description		Source
*Streptomyces* sp. SCSIO 02999		
wild-type	A marine-derived *Streptomyces* sp., cultured in AM6 medium	[37]
*E. coli* K-12 BW25113 strains		
wild-type	*lacI*^q^ *rrnB*_T14_ Δ*lacZ*_WJ16_ *hsdR*514 Δ*araBAD*_AH33_ Δ*rhaBAD*_LD78_ *rph*-1	[68]
Δ*lon*	Δ*lon* Δ km^R^	[68]
BL21(DE3)	F*^−^ompT hsdS_B_(r_B_^−^m_B_^−^) gal dcm* λ(DE3) Ω P_tacUV5_::T7 polymerase	Novagen
WM3064	*thrB*1004 *pro thi rpsL hsdS lacZ*ΔM15 RP4-1360) Δ(*araBAD*)567 Δ*dapA*1341::[*erm pir(*wt)]	Metcalf, W.; UIUC
Plasmids		
pCA24N	Cm^R^; *lacI*^q^	[69]
pCA24N-*vapB*	Cm^R^; *lacI*^q^, P*_T5-lac_*::*vapB*	this study
pCA24N-*vapC*	Cm^R^; *lacI*^q^, P*_T5-lac_*::*vapC*	this study
pCA24N-*vapB-vapC*	Cm^R^; *lacI*^q^, P*_T5-lac_*::*vapB-vapC*	this study
pCA24N-*vapCD6A*	Cm^R^; *lacI*^q^, P*_T5-lac_*::*vapCD5A*, mutant the 5^th^ aa of VapC from D to A	this study
pCA24N-*vapCD96A*	Cm^R^; *lacI*^q^, P*_T5-lac_*::*vapC*, mutant the 95^th^ aa of VapC from D to A	this study
pCA24N-*vapCD114A*	Cm^R^; *lacI*^q^, P*_T5-lac_*::*vapC*, mutant the 113^th^ aa of VapC from D to A	this study
pET28b	Km^R^, *lacI*^q^	-
pET28b-*vapB-vapC*-CHis	Km^R^, *lacI*^q^, pET28b P*_T7-lac_*:: *vapB-vapC* with VapC *C*-terminal His-tagged	this study
pET28b-*vapB-vapC*	Km^R^, *lacI*^q^, pET28b P*_T7-lac_*:: *vapB-vapC* without His-tag	this study
pHGEI01	KmR, R6K *ori*, pHGC01 containing the full-length *E. coli lacZ* gene	[70]
pHGEI01-*vapB*’	pHGEI01 containing the *Streptomyces* sp. native promoter and inactive VapB *N*-termianl 15aa	this study
pHGEI01-*vapC’*	pHGEI01 containing the *Streptomyces* sp. native promoter and *VapB* inactive *VapC N*-termianl 15aa	this study
pHGEI01-*vapB-vapC*	pHGEI01 containing the *Streptomyces* sp. native promoter and *vapB and vapC*	this study

R: resistance, q: quantity, and UIUC standards for University of Illinois at Urbana-Champaign.4.2. Cloning of Genes

## Supplementary Materials

The following are available online at www.mdpi.com/2072-6651/8/7/195/s1, Figure S1: Gene and protein sequence of *vapBC* operon in *Streptomyces* sp. *SCSIO 02999*. Figure S2: VapC in *Streptomyces sp.* SCSIO 02999 belongs to the PIN domain superfamily. Figure S3: VapC mutant could not induce “bubble forming like” cells. Table S1: Oligonucleotides used for plasmid construction site-directed mutagenesis (target mutated nucleotides are in red font and highlighted yellow) and DNA sequencing. If an enzymatic restriction site is included in the sequence, the enzyme restriction site is underlined. f indicates forward primer and r indicates reverse primer. P indicates promoter.
