# Supplementary material for: Characterization of the Deep-Sea Streptomyces sp. SCSIO 02999 Derived VapC/VapB Toxin-Antitoxin System in Escherichia coli
Source: Toxins (Basel). 2016 Jul 1;8(7):195. doi: 10.3390/toxins8070195 (PMC4963828; doi:10.3390/toxins8070195)
Supplement: Supplementary file 1 [file toxins-08-00195-s001.pdf]

Supplementary Materials: Characterization of the Deep-Sea *Streptomyces* sp. SCSIO 02999 Derived VapC/VapB Toxin-Antitoxin System in *Escherichia coli*

Yunxue Guo, Jianyun Yao, Chenglong Sun, Zhongling Wen and Xiaoxue Wang

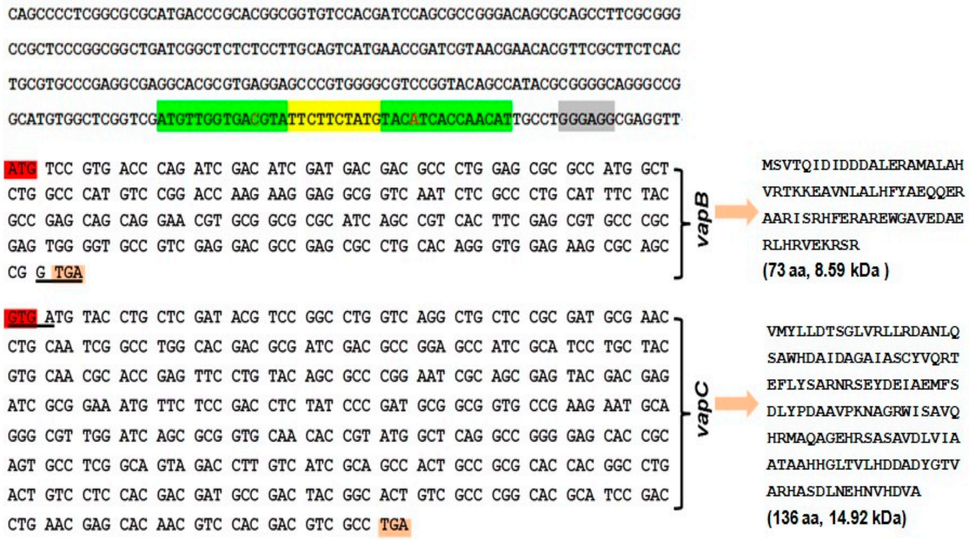

Figure S1. Gene and protein sequence of *vapBC* operon in *Streptomyces* sp. *SCSIO 02999*. The sequence encoding *vapB* and *vapC* are shown as indicated, and the protein sequences of VapB and VapC are also shown together with length and size. The 288 bp of *vapB* 5′ UTR (untranslated regions) is also shown; the palindrome is highlighted, the green indicates the palindrome (14 bp), the yellow indicates the gap, and the mismatch bases are shown in red letters. The ribosome binding site (RBS) is highlighted in gray. The start and stop codons for *vapB* and *vapC* are highlighted in red and orange, respectively. The overlapped four bases are underlined.

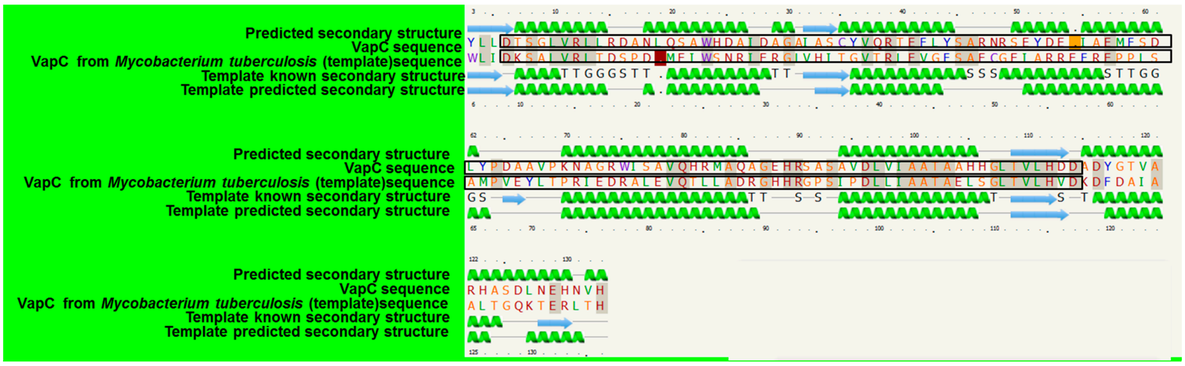

Figure S2. VapC in *Streptomyces* sp. SCSIO 02999 belongs to the PIN domain (PilT *N*-terminal) superfamily. The secondary structure of VapC was predicted using the online PHYRE2 (Protein Homology/analogY Recognition Engine V 2.0) server [1]. The α-helices and β-sheets are shown. VapC was predicted to have a similar secondary structure with several VapC toxins of VapC/VapB TA family. Here, 130 residues (96% of VapC) have been modeled with 100.0% confidence by the known secondary structure of VapC from *Mycobacterium tuberculosis* with the single highest scoring template. The putative active sites (PIN domain) are boxed in open frames.

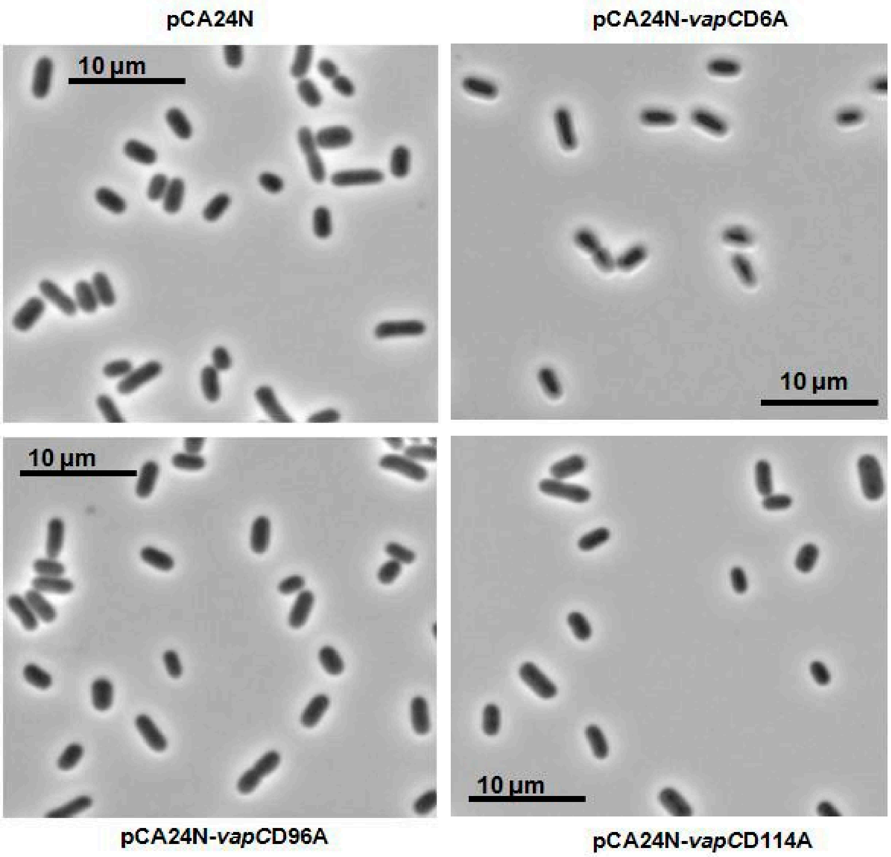

Figure S3. VapC mutant could not induce "bubble forming like" cells. Cells harboring pCA24N, pCA24N-*vap*CD6A, pCA24N-*vap*CD96A, and pCA24N-*vap*CD114A, respectively, were induced with 0.5 mM IPTG at OD_{600} 1.0 for 5 h, and morphology of cells were observed under microscope.

Table S1. Oligonucleotides used for plasmid construction site-directed mutagenesis (target mutated nucleotides are in red font and highlighted yellow) and DNA sequencing. If an enzymatic restriction site is included in the sequence, the enzyme restriction site is underlined. f indicates forward primer and r indicates reverse primer, while P indicates promoter.

Purpose/Name	Sequence (5′-3′)
**Plasmid construction**	
pCA24N-*vapB*-f	GCCTCCGTAACCCAGATCGACAT
pCA24N-*vapB*-r	CCACGGCTGCGCTTCTCCACCCT
pCA24N-*vapC*-f	GCCATGTACCTGCTCGATACGTC
pCA24N-*vapC*-r	CCGGCGACGTCGTGGACGTTGTG
pET28b-*vapB*-f	CTAGCCATGGGCATGTCCGTGACCCAGATCGACAT
pET28b-*vapB*-Chis-r	CCCAAGCTTCTAGTGGTGGTGGTGGTGGTGCCGGCTGCGCTTCTCCACCCTGTGCAGGC
pET28b-*vapC*-f	CTAGCCATGGGCATGTACCTGCTCGATACGTCCGG
pET28b-*vapC*-Chis-r	CCCAAGCTTCTAGTGGTGGTGGTGGTGGTGGGCGACGTCGTGGACGTTGTGCTCGTTCA
pET28b-*vapB-vapC*-r	CCCAAGCTTCTAGGCGACGTCGTGGACGTTGTGCTCGTTCA
pHGEI01-P*vapB-vapC-f*	CCGGAATTCGTCCACGATCCAGCGCCGGGACAG
pHGEI01-*vapB′*-r1	AGCTGTTTCCTGTGTGAGATCTTTACAGAGCCATGGCGCGCTC
pHGEI01-*vapC′*-r1	AGCTGTTTCCTGTGTGAGATCTTTAGGCGTCGATCGCGTCGTG
pHGEI01-*vapB-vapC*-r1	AGCTGTTTCCTGTGTGAGATCTTCAGGCGACGTCGTGGACGTTGTG
pHGEI01-*vapB-vapC*-common-r2	CGCGGATCCGTAATCATGGTCATAGCTGTTTCCTGTGTGAGATCT
**PCR and DNA sequencing**	
pCA24N-f	GATAACAATTTCACACAGAATT
pCA24N-r	GTCAGAGGTTTTCACCGTCATCA
pET28b-f	TAATACGACTCACTATAGGG
pET28b-r	TATGCTAGTTATTGCTCAG
**P*vapB-vapC*-f**	**TTCTCCAGCCCCTCGGCGCGCATGA**
P*vapB-vapC*-r	CATAACCTCGCCTCCCAGGCAATGTTGGTG
**QRT-PCR**	
yoeB-f	AGAACGCCATTTGAAGGTAAGG
yoeB-r	TGAGCAGTGAATCGTCGGTAAC
yefM-f	TGGAGAGGCTTGTGTTCTGATG
yefM-r	TTTCCGTTCCTTTGCCTGAT
relE-f	CACTAAAGGAATGGCGAAAGCT

relE-r	CCAACAGAAATCACGAAAACGA
relB-f	GGTAGCATTAACCTGCGTATTG
relB-r	AGCCGTTCTTTCACTATCTCCAC
mazF-f	TATGGGCGATCTGATTTGGG
mazF-r	TTTCTTCGTTGCTCCTCTTGC
mqsR-f	CACATACACGTTTGAGTCAGGTTAA
mqsR-r	ATCAGAGTAGGTGGTCATGCTTTT
higB-f	AACATAAAACGGAGTTGGTGGC
higB-r	ACGATGAACAGCGGTAAAGAAA
hicA-f	AATCTCAGGGCGTCGATGTAG
hicA-r	CGAGTTGTTTCAGGATTGCTTTA
yafQ-f	ATCAATAATACTTTACCGCTTCCA
yafQ-r	TTATCGGTAAGTTTGTAAATCAGGA
yafO-f	TTTCCTATAAGCGTGACGGTGTT
yafO-r	GAGGTTCAGGTTTCAGAATGGC
yhaV-f	ATCACGGTCAATCCATCATCAC
yhaV-r	GCTGAATACGGTATAGGCATCTGT
***chpB*-f**	**GTTCAAGCCTTTAATCAACTGGG**
chpB-r	TAATAACGCCTCTTCCACCACC
rbn-f	AGTCGCGGCCATAGCTCTAC
rbn-r	GAAATCATTCGCCAGTTCAGTC
ralR-f	CATCAGTAACGGTGAAAGCCA
ralR-r	CCAGTGGTTCGTTTATTCCA
ghoT-f	CCTTTGTCATTATCTGGTTTATCTCAC
ghoT-r	AAAGAGAGAAAAAAGTAATGCCACAG
purA-f	GGGCCTGCTTATGAAGATAAAGT
purA-r	TCAACCACCATAGAAGTCAGGAT
rrsG-f	TATTGCACAATGGGCGCAAG
rrsG-r	ACTTAACAAACCGCCTGCGT
**Single Amino Acid Substitution**	
*vap*CD6A-f	CATGTACCTGCTCGCTACGTCCGGCCTGG
*vap*CD6A -r	CCAGGCCGGACGTAGCGAGCAGGTACATG
*vap*CD96A -f	TGCCTCGGCAGTAGCCCTTGTCATCGCAG
*vap*CD96A -r	CTGCGATGACAAGGGCTACTGCCGAGGCA

*vap*CD114A -f	GTCCTCCACGACGCTGCCGACTACGGC
*vap*CD114A -r	GCCGTAGTCGGCAGCGTCGTGGAGGAC

Reference

1. Kelley, L.A.; Sternberg, M.J. Protein structure prediction on the web: A case study using the phyre server. *Nat. Protoc.* **2009**, *4*, 363–371.
